# Are Patients With Autoimmune Cytopenias at Higher Risk of COVID-19 Pneumonia? The Experience of a Reference Center in Northern Italy and Review of the Literature

**DOI:** 10.3389/fimmu.2020.609198

**Published:** 2021-01-26

**Authors:** Wilma Barcellini, Juri Alessandro Giannotta, Bruno Fattizzo

**Affiliations:** ^1^ Hematology Unit, Fondazione IRCCS Ca’ Granda Ospedale Maggiore Policlinico, Milan, Italy; ^2^ Department of Oncology and Oncohematology, University of Milan, Milan, Italy

**Keywords:** COVID-19, warm autoimmune hemolytic anemia, cold agglutinin disease, immune thrombocytopenia, aplastic anemia, chronic idiopathic neutropenia

## Abstract

During COVID-19 pandemic the care of onco-hematologic and autoimmune patients has raised the question whether they are at higher risk of infection and/or worse outcome. Here, we describe the clinical course of COVID-19 pneumonia in patients with autoimmune cytopenias (AIC) regularly followed at a reference center in Northern Italy. The study period started from COVID-19 outbreak (February 22, 2020) until the time of writing. Moreover, we provide a review of the literature, showing that most cases reported so far are AIC developed during or secondary to COVID-19 infection. At variance, data about AIC pre-existing to COVID infection are scanty. The 4 patients here described (2 autoimmune hemolytic anemias, AIHA, 1 Evans syndrome, and 1 immune thrombocytopenia) with COVID-19 pneumonia belong to a large cohort of 500 AIC patients, making this study nearly population-based. The observed frequency (4/501; 0.7%) is only slightly superior to that of the general population admitted to hospital/intensive care unit (0.28/0.03%, respectively) in Lombardy in the same period of observation. All cases occurred between March 21 and 25, whilst no more AIC were recorded later on. Although different in intensity of care needed, all patients recovered from COVID-19 pneumonia, with apparently no detrimental effect of previous/current immunomodulatory treatments. AIHA relapse occurred in two patients, but promptly responded to therapy. With limitations due to sample size, these results suggest a favorable outcome and a lower-than-expected incidence of COVID-19 pneumonia in patients with previously diagnosed AIC, and allow speculating that immunomodulatory drugs used for AIC may play a beneficial rather than a harmful effect on COVID-19 infection.

## Introduction

COVID-19 pandemic has raised several concerns regarding patients with oncologic, onco-hematologic, and immune-mediated inflammatory/rheumatologic diseases (1–8). On one hand, both disease-related and therapy-induced immunosuppression may increase the risk to contract COVID-19 infection and complicate its management. On the other hand, most of the therapies that have shown some efficacy in COVID-19 infection include drugs with documented immunomodulatory activities, such as steroids, hydroxychloroquine (HCQ), tocilizumab (anti-interleukin 6 receptor), anakinra (anti-interleukin 1 receptor) (9, 10), and complement inhibitors (11), several of them currently employed in various autoimmune diseases. The rationale of their use in COVID-19 infection resides in reducing the exaggerated immune activation and consequent thrombo-inflammation that plays a major role in the unfavorable course of the disease (12). Several case reports described the occurrence of autoimmune hemolytic anemia (AIHA) or immune thrombocytopenia (ITP) developing after or concomitantly to COVID-19 infection (13, 14). At variance, data are lacking about the prevalence and clinical course of COVID-19 infection (asymptomatic or clinically overt) in patients already diagnosed with autoimmune cytopenias (AIC), including AIHA, ITP, chronic idiopathic neutropenia (CIN), and aplastic anemia (AA). Here we describe 4 patients with COVID-19 pneumonia identified among a large cohort of AIC regularly followed at a tertiary university center in Milan, Lombardy, from the outbreak (February 22, 2020) until the time of writing. Furthermore, we critically analyzed the reports of AIC in COVID-19 infection available in literature.

## Materials and Methods

Patients with AIC and COVID-19 pneumonia included one warm AIHA (wAIHA), one cold agglutinin disease (CAD), one ITP and one Evans syndrome (wAIHA plus ITP). They belong to a cohort of 501 AIC actively followed at Hematology Unit of IRCCS Ca’ Granda Ospedale Maggiore Policlinico, Milan, Italy. Clinical, laboratory and radiologic data of patients were collected retrospectively from clinical charts; patients with AIC who developed COVID-19 infection were prospectively followed from February 22, 2020 (time of the first SARS-CoV-2 infection in Lombardy, Italy) until the time of writing. Diagnoses of AIC were made according to international guidelines (15–18). Diagnosis of COVID-19 pneumonia was based on positive nasopharyngeal swab (RT-PCR) and typical radiologic findings of bilateral interstitial pneumonia (19). The study was approved by the local Ethical Committee and patients gave informed consent. The study was conducted in accordance with the Declaration of Helsinki. A review of literature about AIC and COVID-19 was performed by searching for indexed articles from February to September 2020 in MEDLINE *via* PubMed and the National Library of Medicine. The research included cases of COVID-19 infection occurring in previously diagnosed AIC and AIC developing at the onset of COVID-19 infection.

## Results

### AIC Cohort

The clinical features of the AIC cohort (wAIHA, CAD, ITP, CIN, and AA) are summarized in **Table 1**. Patients were mostly middle-aged (with a wide range), and females, as already known for AIC. The follow-up preceding COVID-19 infection was quite long, with a median of about 2 years, and some cases have been followed for more than 15–20 years. Almost all patients with wAIHA, CAD, ITP, and AA had been already treated, with some of them up to five therapy lines; at variance, only one patient with CIN received immunosuppression. Previous infections were particularly frequent in AA, although their rate was not negligible in the other conditions; thrombotic events occurred more often in wAIHA.

**Table 1:** Clinical features of patients with autoimmune cytopenias (AIC) and COVID-19 pneumonia.

	wAIHA N = 139	cAIHA N = 108	ITP N = 103	CIN N = 110	AA N = 41
**Demographics**					
Age, years	64 (19–96)	67 (28–89)	51 (20–92)	56 (15–78)	44 (25–90)
Male/female	61/78	36/72	28/75	37/73	21/20
Follow up, months	31 (7–317)	22.8 (3–179)	23 (6–204)	27 (12–189)	25 (12–216)
**Therapies**					
N. of therapy lines	1 (1–4)	1 (0–4)	1 (0–5)	0 (0–1)	1 (0–3)
Steroids, N(%)	139 (100)	68 (63)	93 (91)	–	37 (90)
Rituximab, N(%)	46 (33)	55 (51)	6 (6)	–	–
Immunosuppressors, N(%)	30 (22)	19 (17)	16 (16)	1	–
Splenectomy, N(%)	11 (8)	3 (2.8)	18 (18)	–	–
ESA, N(%)	14 (10)	8 (7)	–	–	–
Bortezomib, N(%)	–	8 (7)	–	–	–
Danazol, N(%)	–	–	5 (5)	–	3 (6)
TRO-RA, N(%)	–	–	28 (28)	–	7 (17)
ATG, N(%)	–	–	–	–	25 (62)
CyA, N(%)	–	–	–	–	29 (70)
HSCT, N(%)	–	–	–	–	4 (9)
**Complications**					
Thrombosis, N(%)	19 (14)	6 (6)	7 (7)	–	2 (5)
Infections, N(%)	12 (9)	8 (7)	2 (2)	8 (7)	8 (20)

Values are expressed as median(range), unless otherwise specified. Infections are registered according to CTCAE criteria v5.0 and only if >G2. w/cAIHA, Warm and cold autoimmune hemolytic anemia; ITP, immune thrombocytopenia; CIN, chronic idiopathic neutropenia; AA, aplastic anemia; ESA, Erythropoiesis stimulating agents; TPO-RA, Thrombopoietin receptor analogue; ATG, anti-thymocyte globulin; CyA, cyclosporine; including only patients with AA; HSCT, hematopoietic stem cell transplant.

### AIC Cases With COVID-19 Pneumonia

**Table 2** shows the most relevant features of COVID-19 infection of our 4 patients. More in detail, patient #1 is a 59-year-old male diagnosed with primary wAIHA in 2018 and previously treated with steroids and rituximab. At the time of pneumonia, he was receiving an experimental phosphoinositide 3-kinase inhibitor, obtaining a complete response (CR). On March 22, he was admitted to a local hospital because of fever and dyspnoea and was diagnosed with bilateral COVID-19 pneumonia, confirmed by a typical CT scan. The experimental drug was stopped, prednisone increased, and HCQ, tocilizumab, and darunavir administered. After one week, due to worsening respiratory distress, he was transferred to ICU and intubated; low molecular weight heparin (LMWH) prophylaxis was instituted, and several antibiotics administered (ampicillin, meropenem, imipenem, and linezolid) due to likely superimposed bacterial infection. Patient’s general conditions ameliorated and on April 8, he was extubated and transferred to sub-intensive care unit (SICU) and shifted to low-flow oxygen support. Gentamicin and vancomycin were necessary because of Enterococcus faecalis hospital-acquired septicemia. After the resolution of pneumonia, the patient developed an acute bilateral flaccid paraparesis of lower limbs. Brain MRI showed oedema involving posterior parasagittal parietal cortex, with aspects of cerebritis and/or vasculitis. Blood tests for infective causes and autoantibodies for vasculitides resulted negative. These findings were interpreted as dysimmune encephalitis secondary to COVID-19 infection. High-dose intravenous immunoglobulins (IvIg, 0.4 g/kg/day for 5 days) were administered with a slow amelioration of symptoms and disappearance of MRI alterations, allowing discharge after 122 days of hospitalization. During all this period, wAIHA has been in remission, without transfusion requirement.

**Table 2:** Clinical features of patients with autoimmune cytopenias (AIC) and COVID-19 pneumonia.

	Patient #1	Patient #2	Patient #3	Patient #4
**Age, years/gender**	59/M	90/M	78/M	71/F
**Type of AIC**	wAIHA	ITP	Evans syndrome	CAD
**Previous treatments for AIC**	Steroids, rituximab	Steroids, IvIg	Steroids, IvIg, cyclophosphamide, rituximab	Steroids, rituximab
**Ongoing treatments for AIC**	Seroids, experimental phosphoinositide 3-kinase inhibitor	Steroids	Steroids, experimental spleen tyrosine kinase inhibitor	Steroids
**Comorbidities**	Hypertension, asthma, obesity	None	Hypertension, previous myocardial infarction, stroke, septic shocks, and osteonecrosis of the femoral head	Hypertension, osteoporosis
**Date of diagnosis of COVID-19 pneumonia**	March 22, 2020	March 24, 2020	March 25, 2020	March 21, 2020
**Type of admission**	ICU	sub-ICU	Internal medicine ward	Internal medicine ward
**Main clinical/laboratory findings**	Worsening anemia (Hb 8–8.5 g/dL), no transfusion requirement; lymphopenia (nadir 0.24 x10^9/L); high LDH (1,156 U/L), ferritin (4,120 mcg/L) and D-dimer (48,870 mcg/L)	Mild anemia (Hb 10 g/dL), lymphopenia (nadir 0.3 x10^9/L), increased ferritin (4,023 mcg/L), D-dimer (1,864 mcg/L), and fibrinogen (602 mg/dL), and prolonged PT (1.27)	Worsening anemia (Hb 7.3 g/dL), lymphopenia (nadir 0.85 x10^9/L), slightly increase of ferritin levels (445 mcg/L), and prolonged PT (1.45)	Worsening anemia (Hb 5.5 g/dL), lymphopenia (nadir 1 x10^9/L), increased ferritin (1,328 mcg/L), and D-dimer (589 mcg/L)
**treatments**	Intubation, steroids, hydroxychloroquine, tocilizumab, darunavir, and LMWH prophylaxis; IvIg (for post-COVID-19 dysimmune encephalitis)	CPAP, steroids, hydroxychloroquine, and full-dose LMWH	Low-flow oxygen, steroids, hydroxychloroquine, azithromycine, and full-dose LMWH	High-flow oxygen, hydroxychloroquine, azithromycine, lopinavir/ritonavir, steroids, transfusions
**COVID-19 outcome**	Resolved	Dead for superimposed bacterial pneumonia	Resolved	Resolved
**AIC outcome**	Remission	Remission	Remission	Remission

Diagnosis of COVID-19 pneumonia was based on positive nasopharyngeal swab (RT-PCR) and typical radiologic findings of bilateral interstitial pneumonia. wAIHA, warm autoimmune hemolytic anemia; ITP, immune thrombocytopenia; Evans syndrome, AIHA plus ITP; CAD, cold agglutinin disease; ICU, intensive care unit; LMWH, low molecular weight heparin; CPAP, continuous positive airway pressure; IvIg, high dose intravenous immunoglobulins.

Patient #2 is a 90-year-old male diagnosed with ITP in 2011, successfully treated with steroids +/- IvIg and in persistent remission since 2013. On March 5, he was admitted for ITP relapse and treated with steroids and IvIg obtaining complete response. After discharge he was re-admitted to SICU (March 24) for a moderate COVID-19 pneumonia (probably contracted during previous admission). Treatment included continuous positive airway pressure ventilation, HCQ, full-dose LMWH, and continuing full-dose steroids. COVID-associated pneumonia resolved, and the patient continued steroid tapering with persistent ITP remission. Subsequently, the patient was transferred to the general medicine ward and contracted hospital-acquired bacterial pneumonia that required several antibiotics. However, on July 15, a methicillin-sensible Staphilococcus aureus septicemia developed and the patient died from multi organ failure.

Patient #3 is a 78-year-old male with relapsed/refractory Evans’ syndrome diagnosed in 2010. Previous treatments included various cycles of steroids and IvIg, cyclophosphamide, 2 cycles of rituximab. In August 2019, he has been enrolled in a clinical trial with an experimental spleen tyrosine kinase inhibitor for AIHA relapse. His past medical history consisted of arterial hypertension, previous myocardial infarction with ventricular fibrillation, stroke, two septic shocks, and osteonecrosis of the femoral head. On March 25, 2020, he presented at the outpatient clinic with typical symptoms of COVID-19 pneumonia (fever, dyspnoea, desaturation to 80%). He was intercepted by the preventive measures adopted at our hematology department (anamnesis, temperature measurement, swabs) (20). The experimental drug was interrupted, and he was admitted to the internal medicine ward. Pneumonia required low-flow oxygen support, steroids, HCQ, azithromycine, full-dose LMWH, and empirical antibiotic therapy for superimposed bacterial infection. The patient rapidly recovered from pneumonia but experienced two complications: paroxysmal atrial fibrillation treated with amiodarone, and wAIHA relapse that required IvIg and full-dose steroid (prednisone 1 mg/kg/day for 3 weeks followed by slow tapering, still ongoing). After discharge, the study drug was resumed on June 22, and AIHA was in remission at the time of writing.

Patient #4 is a 71-year-old female diagnosed with CAD in 2015 previously treated with steroids and rituximab with complete response. Comorbidities included arterial hypertension and osteoporosis. On March 21, 2020, she was admitted to the internal medicine ward of a local hospital with typical features of SARS-CoV-2 pneumonia and CAD relapse (Hb 5.5 g/dL) requiring transfusions and full-dose steroids. COVID-19 infection was treated with high-flow oxygen, HCQ, azithromycine, lopinavir/ritonavir, and ceftriaxone for superimposed bacterial pneumonia. The patient was discharged after 4 weeks with Hb 9.6 g/dL. Subsequently, hemoglobin rapidly improved, allowing quick tapering of steroids. At the time of writing, blood counts showed normal Hb levels.

### COVID-19 Pandemic in Italy and Epidemiological Considerations

**Figure 1:**
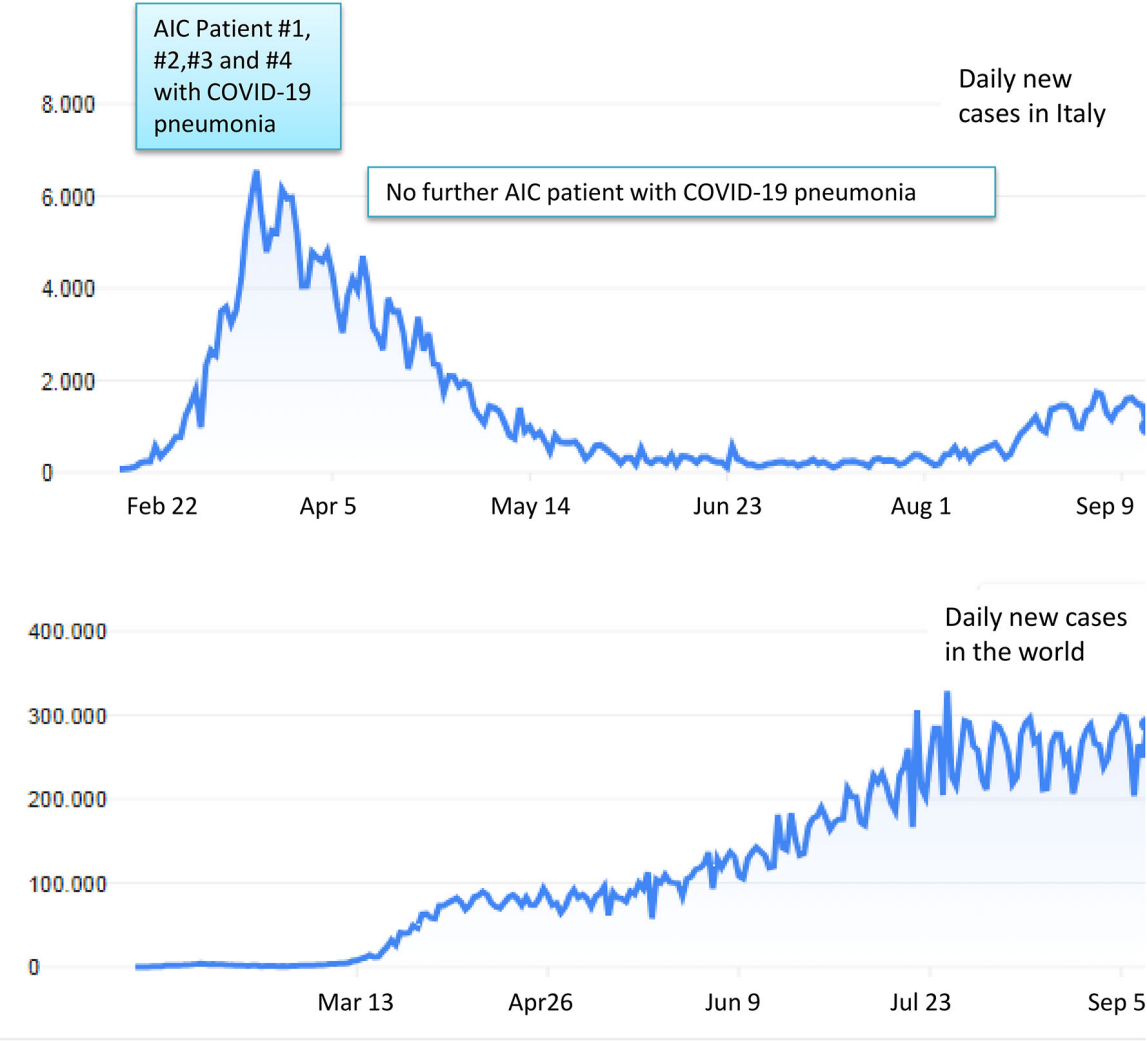
Trends of COVID-19 contagions in Italy (upper panel) and worldwide (lower panel). The four patients with AIC developing COVID-19 pneumonia at our Center were all registered between the end of February and the beginning of April 2020, in coincidence with contagions’ peak in our Country.

The abrupt outbreak of COVID-19 infection began in Lombardy, Northern Italy, on February 22, 2020, with the first patient developing severe pneumonia and ICU admission. In the following weeks, an exponential increase of cases was recorded with a peak at the end of March 2020, and a subsequent progressive decline at the end of May 2020. This curve is quite different for that observed in other countries, where the pandemic developed thereafter and is still ongoing (**Figure 1**). Moreover, about 40% of all Italian COVID-19 cases (42,161/101,739) at the pandemic peak have been recorded in Lombardy. Thereafter this percentage showed no modification, as other regions of Italy maintained a low/absent burden of COVID-19 infection. Among the 42,161 cases, 11,815 and 1,330 were admitted to hospital and ICU, respectively, with an overall mortality around 15% (21, 22). The peculiar setting of our tertiary center in Lombardy, which is a reference center for benign hematology with dedicated Unit and staff, allows some epidemiologic speculations. The size and the characteristics of the population with AIC are representative of disease epidemiology/clinical features, and patients have been regularly followed in the months preceding and during the pandemic, suggesting that our findings may be nearly population-based. The frequency of COVID-19 pneumonia cases within our cohort of AIC (4/501; 0.7%) was only slightly superior to that of the general population admitted to hospital/ICU in Lombardy at the date of March 30 (11,815/1,330 out of 42,161; 0.28/0.03%, respectively for wards and ICU) (21). After this date and until the time of writing no further cases of COVID-19 pneumonia requiring hospital admission were observed among the cohort of AIC patients.

### Literature Review

We considered two settings: COVID19 infection occurring in previously diagnosed AIC (**Table 3**), that is more similar to our study, and AIC developing after or concomitant to COVID19 infection (**Table 4**). Regarding the first, there are few cases reported: 2 Evans syndrome (23, 24) and 2 AA patients (25, 26) who experienced COVID-19 infection, with variable clinical course and outcome (2 resolved, 1 died, and 1 not reported). Other patients with various autoimmune disorders (systemic lupus erythematosus, rheumatoid arthritis, psoriatic arthritis, ankylosing spondylitis, anti-phospholipid syndrome, inflammatory bowel disease) experienced COVID-19 infection, generally with a mild course (5–7, 44). In particular, Haberman et al. reported a prospective case series of 86 patients with immune-mediated inflammatory disease who had either confirmed (59 patients) or highly suspected (27 patients) symptomatic COVID-19 infection, 72% on therapy with biologics or Janus kinase inhibitors. Patients requiring hospitalization (16%, comparable to general COVID-19 infected population), were older and more comorbid than outpatients, and mainly suffered from rheumatoid arthritis. Two patients suffered from more severe disease (1 died) and neither of them was receiving biologic therapies. A recent Italian survey (44) identified 148 symptomatic cases among a series of 916 patients with autoimmune diseases. Symptoms were mainly mild and similar to the general population (cough, sore throat, fever, arthromyalgias, diarrhoea, conjunctivitis, and ageusia/hyposmia) and no deaths were observed. Importantly, only 3% of patients withdrew a medication, mainly immunosuppressants or biologics, due to COVID-19 infection, and >90% of patients declared the adoption of social distancing (36.6% adopted it before official lockdown). In both studies, the Authors concluded that the incidence and severity of COVID-19 were similar to those of the general population, and that the baseline use of biologics was not associated with worse outcomes.

**Table 3:** Literature review of COVID-19 infection occurring in previously diagnosed autoimmune cytopenias (AIC).

AIC type	N° of patients	COVID outcome	AIC Outcome	Reference
Evans syndrome (AIHA)	1 (pediatric)	Resolved	On treatment with MMF and TPO-ra for chronic refractory ITP. Onset of AIHA IgG+C during COVID. Treated with steroids (response).	Wahlster et al. (23)
Evans syndrome	1	Onset with pneumonia and pulmonary embolism (PLT 131 x10^9/L). COVID outcome not described.	Onset during pregnancy. Treated with dexamethasone + IvIg and then rituximab (ongoing at COVID infection) for refractory ITP.	Vadlamudi et al. (24)
AA	1 (pediatric)	Resolved	Treated with supportive care as bridge to allogeneic MUD-HSCT.	Akcabelen et al. (25)
AA	1	Died for COVID-associated acute necrotizing encephalopathy	Previously treated with immunosuppressive therapy. Treated with supportive care during COVID infection.	Dixon et al. (26)

IvIg, intravenous immunoglobulins; TPO-ra, thrombopoietin receptor analogue; ITP, immune thrombocytopenia; AIHA, autoimmune hemolytic anemia; AIHA IgG+C, warm type AIHA with Coombs test positive for IgG and complement fraction; AA, aplastic anemia; MUD-HSCT, matched unrelated donor hematopoietic stem cell transplant.

**Table 4:** Literature review of autoimmune cytopenias (AIC) developing after or concomitant to COVID-19 infection.

AIC type	N° of patients	COVID outcome	AIC Outcome	Reference
ITP	3	Resolved in two patients. One died for intraventricular hemorrhage + pulmonary embolism + pneumonia.	Treated with IvIg + steroids with response (n = 2) Refractory to platelet pools (n = 1).	Bomhof et al. (27)
ITP	1 (pediatric)	Resolved	Responded to IvIg	Tsao et al. (28)
ITP	1	Resolved	Responded to IvIg	Murt et al. (29)
ITP	1	Resolved	Treated with high-dose steroids, IvIg, romiplostim, vincristine, with complete response.	Lévesque et al. (30)
ITP	1	Resolved	Responded to IvIg + steroids	Artru et al. (31)
ITP	1	Resolved	Responded to IvIg. At relapse responded to IvIg + steroids.	Bennett et al. (32)
ITP	14	Resolved	Treated with IvIg (n = 9); steroids (n = 8); TPO-ra (n = 4). Complete response in n = 11; relapse in n = 3.	Mahévas et al. (14)
ITP	1	Resolved	Failed IvIg, responded to steroids	Hindilerden et al. (33)
AIHA	7	Resolved	N = 4 wAIHA (previous untreated CLL n = 2, IgG-MGUS n = 1) N = 3 cAIHA (n = 2 concomitant MZL diagnosis, n = 1 concomitant prostate cancer diagnosis). Treated with steroids (n = 6), and rituximab (n = 2) for steroid-refractoriness.	Lazarian et al. (13)
wAIHA IgG+C	1	Resolved	Responded to steroids + IvIg	Lopez et al. (34)
AIHA IgG+C	1	Resolved	Responsive to IvIg + steroid	Hindilerden et al. (35)
wAIHA	1 (pediatric)	Resolved	Responded to steroid	Vega Hernández et al. (36)
cAIHA and mixed type AIHA	2	Resolved	Resolved after transfusions	Huscenot et al. (37)
cAIHA	1	Resolved (concomitant positive IgM serology for Mycoplasma pneumoniae)	Spontaneous resolution	Moonla et al. (38)
cAIHA	1	Resolved	Resolved after transfusions	Capes et al. (39)
cAIHA	1	Resolved (onset with pulmonary embolism)	Spontaneous resolution	Patil et al. (40)
cAIHA	1	Dead (onset with lethargia and bilateral upper limb thrombosis)	Transfused. Died after 48 h.	Maslov et al. (41)
Evans syndrome	1	Resolved	ITP responsive to IvIg. AIHA (onset 1 week after) responsive to steroid.	Li et al. (42)
AA	1 (pediatric)	Resolved with convalescent plasma	Persistence after SARS-CoV-2 clearance. Only transfusional and anti-baterial/fungal support.	Figlerowicz et al. (43)

IvIg, intravenous immunoglobulins; TPO-ra, thrombopoietin receptor analogue; ITP, immune thrombocytopenia; AIHA, autoimmune hemolytic anemia; wAIHA, warm type AIHA with Coombs test positive for IgG only; AIHA IgG+C, warm type AIHA with Coombs test positive for IgG and complement fraction; cAIHA, cold agglutinin disease; AA, aplastic anemia; MUD-HSCT, matched unrelated donor hematopoietic stem cell transplant.

Regarding AIC cases reported after or concomitant to COVID-19 infection/pneumonia (**Table 4**), the most represented is ITP, with 23 patients (mostly case reports) that developed COVID-19-associated acute/severe thrombocytopenia. This cytopenia was not present before infection and was different from the frequently observed mild thrombocytopenia accompanying COVID-19 (14, 27–33). All cases resolved with steroids and/or IvIg with the exception of one patient who died for intra-ventricular hemorrhage and pulmonary embolism/pneumonia (27). Few cases required other treatments (TPO-RA in 5 patients, of whom 1 also received vincristine). Sixteen AIHA patients were reported, mostly cold forms, of whom one fatal (13, 34–41). Four cases were associated with previous lymphoproliferative disorders (chronic lymphocytic leukemia and mantle zone lymphoma). Treatments included steroids+/-IvIg in half of cases, and rituximab in two patients. Some cases required only transfusion support and two resolved without specific therapy. Finally, a patient with Evans syndrome (concomitant ITP and AIHA) and a pediatric patient with AA have been described (42, 43), the first resolved with steroids and IvIg, the second recovered from COVID-infection but still required AA treatment.

## Discussion

Here, we describe four patients with AIC who favorably recovered from COVID-19 pneumonia. Many concerns have been raised about COVID-19 incidence and clinical course in patients with autoimmune diseases, especially those on immunomodulatory/immunosuppressant therapy. Haberman et al. first described the characteristics of COVID-19 infection in a large series of patients with systemic autoimmune diseases (5), reporting an incidence comparable to the general population and a favorable outcome in both hospitalized and outpatients. Importantly, baseline use of biologics was not associated with worse COVID-19 outcomes. Contrarily, other reports on rheumatologic patients indicated an increased incidence of COVID-19 in this patients’ population, and pointed out that this frequency may be underestimated due to the known “diagnostic gap” of asymptomatic patients and to some clinical features shared by the two conditions (7). Our experience is more similar to that of Haberman et al., with less than 1% incidence of symptomatic COVID-19 infection and a favorable outcome in the majority of them. However, under-detection of asymptomatic cases cannot be excluded, despite hematologic patients are educated to refer to the Hematology Unit for almost all clinical problems, and we provided active and close follow-up through telemedicine means. Moreover, the preventive measures adopted at our Unit may have contributed to reduce contagions (20). Additionally, autoimmune cytopenias may imply a less severe immune disruption as compared to systemic autoimmune conditions.

A plethora of immunosuppressant drugs have been tested in COVID-19 pneumonia itself, with only dexamethazone showing evidence for better survival (45, 46). Consistently, in our experience the use of immunomodulatory/immunosuppressant drugs, including biologics, did not seem to either increase the incidence or adversely impact on the outcome. In fact, patient #1 experienced a favorable COVID-19 outcome without concurrent wAIHA relapse. This may be also related to aggressive COVID-19 specific therapy, although the effect of the recent AIHA-directed immunomodulation cannot be excluded. Patient #3 had a heavily pre-treated relapsed/refractory Evans syndrome, and a high burden of comorbidities. The clinical course of COVID-19 infection was even more favorable than in the previous case, although wAIHA relapse required treatment with resuming of the experimental drug after recovery from pneumonia. Also, in patient #4, COVID-19 infection was concomitant to severe CAD relapse, possibly triggered by virus-induced hyperactivation of the complement cascade. However, the latter 2 AIC reactivations were not catastrophic and promptly managed. Finally, patient #2, who experienced a milder COVID-19 disease, had a fatal outcome probably more related to older age and comorbidities. Importantly, ITP was in remission and previous immunosuppressive therapy was very limited (steroids only). Notably, no COVID-19 pneumonia was observed among patients with CIN and AA, who are classically considered at risk of infection.

Our COVID-19 pneumonia cases occurred in patients previously diagnosed with AIC. This setting is poorly described in literature for hematologic diseases, at variance with systemic autoimmune/rheumatologic disorders. Most of COVID-related cytopenias described in literature developed at the time of COVID-19 infection (generally within the first month). It is largely known that autoimmune cytopenias may be triggered by viral and bacterial infections, due to mechanisms of molecular mimicry, neo-antigen generation and hidden epitope spreading (47, 48). Interestingly, the RBC membrane protein ankyrin-1 was found to share a 100% identity with the SARS-CoV-2 surface glycoprotein Spike (49), suggesting a molecular mimicry mechanism for AIHA. Regarding ITP, anti-GP IIb/IIIa, GP-Ib/IX, or GP-V antibodies have been identified in several cases (50), although a sequence homology between SARS-CoV-2 and platelet components still needs to be documented. Furthermore, during infections, platelets and viruses interact in a sialic acid–dependent manner, leading to increased hepatic clearance of platelets. Sialic acids may act as additional receptors for SARS-COV-2 spike protein, possibly accounting for thrombocytopenia in COVID-19, as observed for influenza virus (51–53). Finally, AIC secondary to infections are thought to be often transient and/or promptly responsive to first-line therapy. Accordingly, the literature review showed that some cases of AIC secondary to COVID-19 resolved spontaneously and most responded to frontline steroid therapy. In the acute setting, the differential diagnosis of AIC may be challenging, since COVID infection itself is characterized by a variable degree of cytopenia as well as immunoregulatory cytokine storm (54). This is particularly true for thrombocytopenia, whose autoimmune origin is difficult to establish (14). Although our Unit is a reference center for autoimmune cytopenias and Lombardy was heavily hit by COVID-19 infection, we did not observe nor were notified of new-onset AIC secondary to SARS-CoV-2 infection. Finally, evidences are accumulating on the immune-disruptive potential of COVID-19 infection. In particular, a very severe onset of systemic lupus erythematous plus anti-phospholipid syndrome (55) and various immune-mediated neurologic sequelae (56) are increasingly described. In our series, the only immune complication secondary to COVID infection was the dysimmune encephalitis in patient #1.

In conclusion, although limited in sample size, our experience shows a lower-than-expected prevalence and favorable outcome of COVID-19 pneumonia in patient with AIC. We may hypothesize that previous/concurrent immunosuppressive treatments, rather than increasing the risk of contagion, may smolder the clinical course of the disease.

## Data Availability Statement

The raw data supporting the conclusions of this article will be made available by the authors, without undue reservation.

## Ethics Statement

The studies involving human participants were reviewed and approved by Comitato Etico Milano Area 2, Fondazione IRCCS Ca’ Granda Ospedale Maggiore Policlinico, via F. Sforza 28, 20100, Milano, Italy. The patients/participants provided their written informed consent to participate in this study.

## Author Contributions

All authors (WB, JAG, and BF) followed patients, wrote and revised the manuscript for important intellectual content. All authors contributed to the article and approved the submitted version.

## Funding

This work was supported by research funding from Fondazione IRCCS Ca’ Granda Ospedale Maggiore Policlinico (RC 2019).

## Conflict of Interest

The authors declare that the research was conducted in the absence of any commercial or financial relationships that could be construed as a potential conflict of interest.

## References

[1] van de Haar J Hoes LR Coles CE Seamon K Fröhling S Jäger D Caring for patients with cancer in the COVID-19 era. Nat Med (2020) 26:665–71. 10.1038/s41591-020-0874-8 32405058

[2] He W Chen L Chen L Yuan G Fang Y Chen W COVID-19 in persons with haematological cancers. Leukemia (2020) 34:1637–45. 10.1038/s41375-020-0836-7 PMC7180672 32332856

[3] El-Sharkawi D Iyengar S Haematological cancers and the risk of severe COVID-19: Exploration and critical evaluation of the evidence to date. Br J Haematol (2020) 190:336–45. 10.1111/bjh.16956 PMC7323194 32559308

[4] Valenza F Papagni G Marchianò A Daidone MG DeBraud F Colombo MP Response of a comprehensive cancer center to the COVID-19 pandemic: the experience of the Fondazione IRCCS-Istituto Nazionale dei Tumori di Milano. Tumori (2020) 106:193–202. 10.1177/0300891620923790 32364028

[5] Haberman R Axelrad J Chen A Castillo R Yan D Izmirly P Covid-19 in Immune-Mediated Inflammatory Diseases - Case Series from New York. N Engl J Med (2020) 383:85–8. 10.1056/NEJMc2009567 PMC7204427 32348641

[6] Tomelleri A Sartorelli S Campochiaro C Baldissera EM Dagna L Impact of COVID-19 pandemic on patients with large-vessel vasculitis in Italy: a monocentric survey. Ann Rheum Dis (2020) 79:1252–3. 10.1136/annrheumdis-2020-217600 PMC7456557 32345617

[7] Ferri C Giuggioli D Raimondo V L’Andolina M Tavoni A Cecchetti R COVID-19 and rheumatic autoimmune systemic diseases: report of a large Italian patients series. Clin Rheumatol (2020) 39:1–10. 10.1007/s10067-020-05334-7 32852623 PMC7450255

[8] Hasseli R Mueller-Ladner U Schmeiser T Hoyer BF Krause A Lorenz HM National registry for patients with inflammatory rheumatic diseases (IRD) infected with SARS-CoV-2 in Germany (ReCoVery): a valuable mean to gain rapid and reliable knowledge of the clinical course of SARS-CoV-2 infections in patients with IRD. RMD Open (2020) 6:e001332. 10.1136/rmdopen-2020-001332 32878994 PMC7507994

[9] Alijotas-Reig J Esteve-Valverde E Belizna C Selva-O’Callaghan A Pardos-Gea J Quintana A Immunomodulatory therapy for the management of severe COVID-19. Beyond the anti-viral therapy: A comprehensive review. Autoimmun Rev (2020) 19:102569. 10.1016/j.autrev.2020.102569 32376394 PMC7252146

[10] Burrage DR Koushesh S Sofat N Immunomodulatory Drugs in the Management of SARS-CoV-2. Front Immunol (2020) 11:1844. 10.3389/fimmu.2020.01844 PMC7438578 32903555

[11] Polycarpou A Howard M Farrar CA Greenlaw R Fanelli G Wallis R Rationale for targeting complement in COVID-19. EMBO Mol Med (2020) 12:e12642. 10.15252/emmm.202012642 32559343 PMC7323084

[12] Connors JM Levy JH Thromboinflammation and the hypercoagulability of COVID-19. J Thromb Haemost (2020) 18:1559–61. 10.1111/jth.14849 PMC9770920 32302453

[13] Lazarian G Quinquenel A Bellal M Siavellis J Jacquy C Re D Autoimmune haemolytic anaemia associated with COVID-19 infection. Br J Haematol (2020) 190:29–31. 10.1111/bjh.16794 32374906 PMC7267601

[14] Mahévas M Moulis G Andres E Riviere E Garzaro M Crickx E Clinical characteristics, management and outcome of Covid-19-associated immune thrombocytopenia. A French multicenter series. Br J Haematol (2020) 190:e224–9. 10.1111/bjh.17024 PMC7404899 32678953

[15] Jäger U Barcellini W Broome CM Gertz MA Hill A Hill QA Diagnosis and treatment of autoimmune hemolytic anemia in adults: Recommendations from the First International Consensus Meeting. Blood Rev (2020) 41:100648. 10.1016/j.blre.2019.100648 31839434

[16] Neunert C Terrell DR Arnold DM Buchanan G Cines DB Cooper N American Society of Hematology 2019 guidelines for immune thrombocytopenia. Blood Adv (2019) 3:3829–66. 10.1182/bloodadvances.2019000966 PMC6963252 31794604

[17] Newburger PE Autoimmune and other acquired neutropenias. Hematol Am Soc Hematol Educ Program (2016) 2016:38–42. 10.1182/asheducation-2016.1.38 PMC5380382 27913460

[18] Killick SB Bown N Cavenagh J Dokal I Foukaneli T Hill A Guidelines for the diagnosis and management of adult aplastic anaemia. Br J Haematol (2016) 172:187–207. 10.1111/bjh.13853 26568159

[19] Blažić I Brkljačić B Frija G The use of imaging in COVID-19-results of a global survey by the International Society of Radiology. Eur Radiol (2020). 10.1007/s00330-020-07252-3 PMC7494433 32939620

[20] Fattizzo B Giannotta JA Barcellini W Barbanti MC Bucelli C Cassin R Ensuring continuity of care of hematologic patients during COVID-19 pandemic in a tertiary hospital in Lombardy (Italy). Blood Adv (2020) 4:2996–9. 10.1182/bloodadvances.2020002120 PMC7362383 32609844

[21] Grasselli G Zangrillo A Zanella A Antonelli M Cabrini L Castelli A Baseline Characteristics and Outcomes of 1591 Patients Infected With SARS-CoV-2 Admitted to ICUs of the Lombardy Region, Italy. JAMA (2020) 323:1574–81. 10.1001/jama.2020.5394 PMC7136855 32250385

[22] COVID-19 Dashboard by the Center for Systems Science and Engineering (CSSE) at Johns Hopkins University (JHU). Available at: https://www.arcgis.com/apps/opsdashboard/index.html#/bda7594740fd40299423467b48e9ecf6 (Accessed May 2, 2020).

[23] Wahlster L Weichert-Leahey N Trissal M Grace RF Sankaran VG COVID-19 presenting with autoimmune hemolytic anemia in the setting of underlying immune dysregulation. Pediatr Blood Cancer (2020) 3:e28382. 10.1002/pbc.28382 PMC7674227 32495391

[24] Vadlamudi G Hong L Keerthy M Evans Syndrome Associated with Pregnancy and COVID-19 Infection. Case Rep Obstet Gynecol (2020) 2020:8862545. 10.1155/2020/8862545 32850163 PMC7441452

[25] Akcabelen YM Koca Yozgat A Parlakay AN Yarali N COVID-19 in a child with severe aplastic anemia. Pediatr Blood Cancer (2020) 67:e28443. 10.1002/pbc.28443 32539221 PMC7323031

[26] Dixon L Varley J Gontsarova A Mallon D Tona F Muir D COVID-19-related acute necrotizing encephalopathy with brain stem involvement in a patient with aplastic anemia. Neurol Neuroimmunol Neuroinflamm (2020) 7:e789. 10.1212/NXI.0000000000000789 32457227 PMC7286661

[27] Bomhof G Mutsaers PGNJ Leebeek FWG Te Boekhorst PAW Hofland J Croles FN COVID-19-associated immune thrombocytopenia. Br J Haematol (2020) 190:e61–4. 10.1111/bjh.16850 PMC7276755 32420612

[28] Tsao HS Chason HM Fearon DM Immune Thrombocytopenia (ITP) in a Pediatric Patient Positive for SARS-CoV-2. Pediatrics (2020) 146:e20201419. 10.1542/peds.2020-1419 32439817

[29] Murt A Eskazan AE Yılmaz U Ozkan T Ar MC COVID-19 presenting with immune thrombocytopenia: A case report and review of the literature. J Med Virol (2020) 4:2048–58. 10.1002/jmv.26138 PMC7300669 32497344

[30] Lévesque V Millaire É Corsilli D Rioux-Massé B Carrier FM Severe immune thrombocytopenic purpura in critical COVID-19. Int J Hematol (2020) 1:1–5. 10.21203/rs.3.rs-32479/v1 PMC7327458 32613314

[31] Artru F Alberio L Moradpour D Stalder G Acute immune thrombocytopaenic purpura in a patient with COVID-19 and decompensated cirrhosis. BMJ Case Rep (2020) 13:e236815. 10.1136/bcr-2020-236815 PMC7342180 32641442

[32] Bennett J Brown C Rouse M Hoffmann M Ye Z Immune Thrombocytopenia Purpura Secondary to COVID-19. Cureus (2020) 12:e9083. 10.7759/cureus.9083 32676257 PMC7362597

[33] Hindilerden F Yonal-Hindilerden I Sevtap S Kart-Yasar K Immune Thrombocytopenia in a Very Elderly Patient With Covid-19. Front Med (Lausanne) (2020) 7:404 a. 10.3389/fmed.2020.00404 32754609 PMC7365892

[34] Lopez C Kim J Pandey A Huang T DeLoughery TG Simultaneous onset of COVID-19 and autoimmune haemolytic anaemia. Br J Haematol (2020) 190:31–2. 10.1111/bjh.16786 PMC7267644 32369626

[35] Hindilerden F Yonal-Hindilerden I Akar E Yesilbag Z Kart-Yasar K Severe Autoimmune Hemolytic Anemia in COVID-19 İnfection, Safely Treated with Steroids. Mediterr J Hematol Infect Dis (2020) 12:e2020053 b. 10.4084/mjhid.2020.053 32670531 PMC7340241

[36] Vega Hernández P Borges Rivas Y Ortega Sánchez E Marqués Cabrero A Remedios Mateo L Silvera Roig P Autoimmune Hemolytic Anemia in a Pediatric Patient With Severe Acute Respiratory Syndrome Coronavirus 2 Infection. Pediatr Infect Dis J (2020) 39:e288. 10.1097/INF.0000000000002809 32639462

[37] Huscenot T Galland J Ouvrat M Rossignol M Mouly S Sène D SARS-CoV-2-associated cold agglutinin disease: a report of two cases. Ann Hematol (2020) 99:1943–4. 10.1007/s00277-020-04129-9 PMC7317069 32591877

[38] Moonla C Watanaboonyongcharoen P Suwanpimolkul G Paitoonpong L Jantarabenjakul W Chanswangphuwana C Cold agglutinin disease following SARS-CoV-2 and Mycoplasma pneumoniae co-infections. Clin Case Rep (2020) 20:723–4. 10.1002/ccr3.3152 PMC7404354 32837721

[39] Capes A Bailly S Hantson P Gerard L Laterre PF COVID-19 infection associated with autoimmune hemolytic anemia. Ann Hematol (2020) 99:1679–80. 10.1007/s00277-020-04137-9 PMC7295688 32542444

[40] Patil NR Herc ES Girgis M Cold agglutinin disease and autoimmune hemolytic anemia with pulmonary embolism as a presentation of COVID-19 infection. Hematol Oncol Stem Cell Ther (2020) S1658-3876:30116–3. 10.1016/j.hemonc.2020.06.005 PMC7336954 32645300

[41] Maslov DV Simenson V Jain S Badari A COVID-19 and Cold Agglutinin Hemolytic Anemia. TH Open (2020) 4:e175–7. 10.1055/s-0040-1715791 PMC7440967 32844144

[42] Li M Nguyen CB Yeung Z Sanchez K Rosen D Bushan S Evans syndrome in a patient with COVID-19. Br J Haematol (2020) 190:e59–61. 10.1111/bjh.16846 PMC7276874 32420629

[43] Figlerowicz M Mania A Lubarski K Lewandowska Z Służewski W Derwich K First case of convalescent plasma transfusion in a child with COVID-19-associated severe aplastic anemia. Transfus Apher Sci (2020) 1:102866. 10.1016/j.transci.2020.102866 PMC7328608 32636116

[44] Zen M Fuzzi E Astorri D Saccon F Padoan R Ienna L SARS-CoV-2 infection in patients with autoimmune rheumatic diseases in northeast Italy: A cross-sectional study on 916 patients. J Autoimmun (2020) 112:102502. 10.1016/j.jaut.2020.102502 32527675 PMC7832807

[45] Favalli EG Ingegnoli F De Lucia O Cincinelli G Cimaz R Caporali R COVID-19 infection and rheumatoid arthritis: Faraway, so close! Autoimmun Rev (2020) 19:102523. 10.1016/j.autrev.2020.102523 32205186 PMC7102591

[46] Horby P Lim WS Emberson JR Mafham M Bell JL Linsell L Dexamethasone in Hospitalized Patients with Covid-19 - Preliminary Report. N Engl J Med (2020). 10.1056/NEJMoa2021436 PMC7383595 32678530

[47] Barcellini W Giannotta J Fattizzo B Autoimmune hemolytic anemia in adults: primary risk factors and diagnostic procedures. Expert Rev Hematol (2020) 13:585–97. 10.1080/17474086.2020.1754791 32274943

[48] Barcellini W Fattizzo B The Changing Landscape of Autoimmune Hemolytic Anemia. Front Immunol (2020) 11:946. 10.3389/fimmu.2020.00946 32655543 PMC7325906

[49] Angileri F Légaré S Marino Gammazza A Conway de Macario E Macario AJL Cappello F Is molecular mimicry the culprit in the autoimmune haemolytic anaemia affecting patients with COVID-19? Br J Haematol (2020) 190:e92–3. 10.1111/bjh.16883 PMC7283741 32453861

[50] Bhattacharjee S Banerjee M Immune Thrombocytopenia Secondary to COVID-19: a Systematic Review. SN Compr Clin Med (2020) 2:1–11. 10.1007/s42399-020-00521-8 PMC7501509 32984764

[51] Baker AN Richards SJ Guy CS Congdon TR Hasan M Zwetsloot AJ The SARS-COV-2 Spike Protein Binds Sialic Acids and Enables Rapid Detection in a Lateral Flow Point of Care Diagnostic Device. ACS Cent Sci (2020) 5:acscentsci.0c00855. 10.1021/acscentsci.0c00855 PMC7523238 33269329

[52] Morniroli D Giannì ML Consales A Pietrasanta C Mosca F Human Sialome and Coronavirus Disease-2019 (COVID-19) Pandemic: An Understated Correlation? Front Immunol (2020) 11:1480. 10.3389/fimmu.2020.01480 32655580 PMC7324714

[53] Jansen AJG Spaan T Low HZ Di Iorio D van den Brand J Tieke M Influenza-induced thrombocytopenia is dependent on the subtype and sialoglycan receptor and increases with virus pathogenicity. Blood Adv (2020) 4:2967–78. 10.1182/bloodadvances.2020001640 PMC7362372 32609845

[54] Tang Y Liu J Zhang D Xu Z Ji J Wen C Cytokine Storm in COVID-19: The Current Evidence and Treatment Strategies. Front Immunol (2020) 11:1708. 10.3389/fimmu.2020.01708 32754163 PMC7365923

[55] Mantovani Cardoso E Hundal J Feterman D Magaldi J Concomitant new diagnosis of systemic lupus erythematosus and COVID-19 with possible antiphospholipid syndrome. Just a coincidence? A case report and review of intertwining pathophysiology. Clin Rheumatol (2020) 39:2811–5. 10.1007/s10067-020-05310-1 PMC7384868 32720260

[56] Najjar S Najjar A Chong DJ Pramanik BK Kirsch C Kuzniecky RI Central nervous system complications associated with SARS-CoV-2 infection: integrative concepts of pathophysiology and case reports. J Neuroinflamm (2020) 17:231. 10.1186/s12974-020-01896-0 PMC7406702 32758257

